# A novel frameshift mutation in the *XPC* gene in a Moroccan patient: a case report

**DOI:** 10.1186/s13256-017-1311-6

**Published:** 2017-06-15

**Authors:** Yassamine Doubaj, Wiam Smaili, Fatima-Zahra Laarabi, Abdelaziz Sefiani

**Affiliations:** 1 Centre de génomique humaine, Faculté de Médecine et de Pharmacie de Rabat, Université Mohammed V, Rabat, Morocco; 2 Département de Génétique Médicale, Institut National d’Hygiène, 27, Avenue Ibn Batouta, BP 769, 11400 Rabat, Morocco

**Keywords:** Xeroderma pigmentosum, *XPC*, Novel mutation, Moroccan

## Abstract

**Background:**

Xeroderma pigmentosum is an autosomal recessive inherited disease. The diagnosis is essentially based on clinical findings and the family history. This genodermatosis is genetically heterogeneous; to date, nine genes have been associated to this disorder. Based on the result of many studies, xeroderma pigmentosum complementation group C is the most common form of xeroderma pigmentosum. A founder mutation in the *XPC* gene was reported in the Maghreb region of northern Africa. According to these findings, the Department of Medical Genetics in Rabat offers molecular diagnosis by screening for the recurrent mutation c.1643_1644delTG which represents 74% of all the probands with xeroderma pigmentosum.

**Case presentation:**

We describe the case of a 21-year-old Moroccan son of consanguineous parents diagnosed with xeroderma pigmentosum on the basis of sun-exposed skin abnormalities and bilateral ocular involvement.

A molecular study led to the identification of a new frameshift insertion of four nucleotides in exon 9.

**Conclusions:**

To the best of our knowledge, this mutation has not been described. The sequencing of the ninth exon should be proposed as first line molecular analysis for all Moroccan patients with xeroderma pigmentosum.

## Background

Xeroderma pigmentosum (XP), (Online Mendelian Inheritance in Man (OMIM) 278700-278780), is a very rare genodermatosis with an estimated prevalence of 1/1,000,000 in United States (USA) and Europe [1] while it is more common in Japan [2] and in other populations with high levels of consanguinity, such as Morocco where the prevalence was estimated to be 1/80,504 [3].

The diagnosis of XP is based essentially on clinical findings with an early onset of skin abnormalities on individuals’ sun-exposed areas during their first years of life. Ocular manifestations limited to the anterior portion are as common as cutaneous lesions. Progressive neurological manifestations are seen in only 25% of affected individuals [4].

The prognosis of the disease is related to neurological degeneration and skin cancer. Patients with XP have an increased risk of cutaneous neoplasia (basal cell carcinoma, squamous cell carcinoma, and melanoma).

XP is genetically heterogeneous with nine complementation groups associated with biallelic mutations in nucleotide excision repair (NER) genes or in the deoxyribonucleic acid (DNA) bypass polymerase *POLH*. In several studies, *XP* complementation group *C* (*XPC*) is the major disease-causing gene with a recurrent mutation in the Mediterranean region [5, 6]. According to these findings, the Department of Medical Genetics in Rabat recommends the molecular diagnosis of XP by screening for the recurrent mutation: c.1643_1644delTG (p.Val548AlafsX25) in the *XPC* gene.

## Case presentation

The proband is a 21-year-old Moroccan man, the sixth child of healthy consanguineous parents of Moroccan descent with no family history. Pregnancy and delivery were normal. He has a normal motor and mental development. When he was 9-months old, skin changes appeared in photo-exposed areas especially on his face. He has been operated twice for bilateral ocular tumors at the age of 15-years old. On clinical examination, he presented photophobia and freckling of the skin on his face, neck, and limbs. He also had bilateral ocular involvement with conjunctival inflammatory lesions spreading to the cornea. There was no evidence of premalignant skin lesions (actinic keratosis) or apparent neurological abnormalities.

**Fig. 1:**
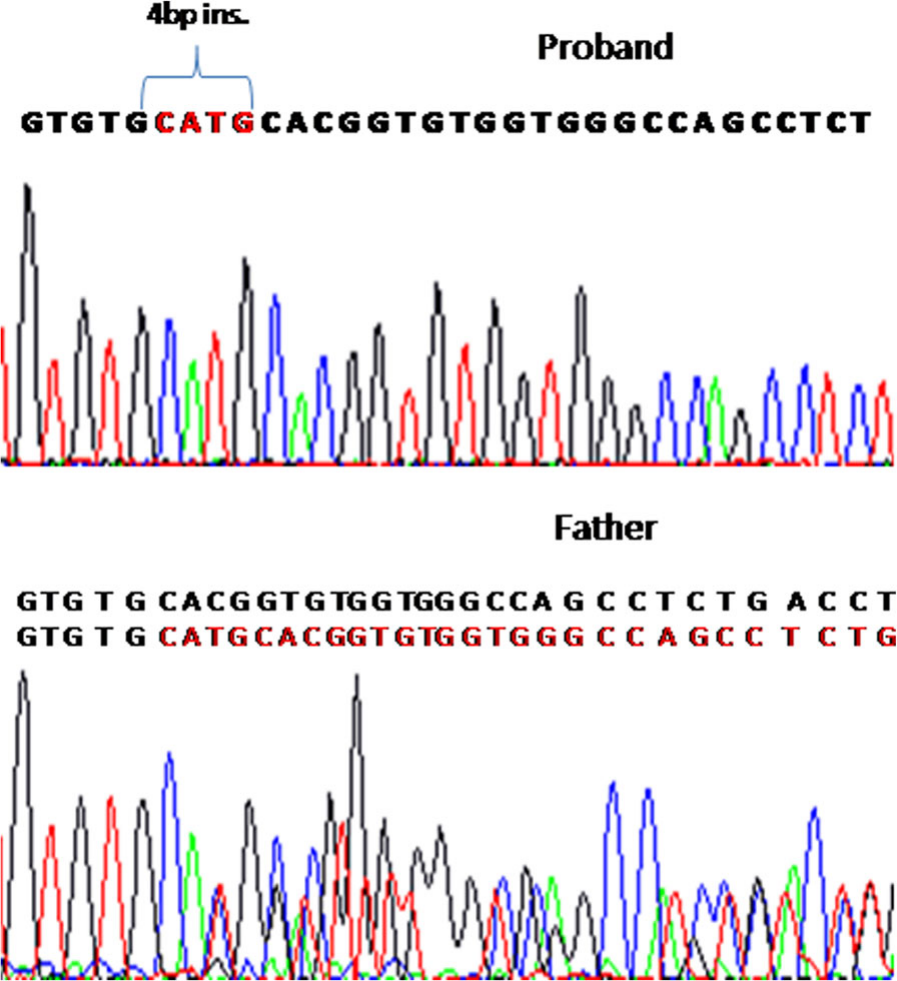
Electrophoregram showing the mutation at an homozygous state in the proband and at an heterozygous state in the father

We collected peripheral blood from him and his parents after an informed consent. DNA was extracted with QIAamp DNA Blood Mini Kit (QiaGen). Molecular genetic testing was performed by amplifying the ninth exon of *XPC* gene by polymerase chain reaction (PCR) to screen for the recurrent mutation, using the following primers: *XPC-*9F: GGCATCCTCAAGCTCTTCAA and *XPC*-9R: GGGCTCTGGTATGGTCTCAA. The PCR protocol included an initial denaturation of 95 °C for 1 minute, followed by 35 cycles of 95 °C for 15 seconds, 61 °C for 15 seconds, and 72 °C for 10 seconds. This led to the identification of a novel homozygous insertion of four bases within the ninth exon of the gene leading to a premature stop codon c.1644_1645insCATG (p.G550Afs*25) (NM_004628). His parents were tested and were found to be heterozygous for the mutation (Fig. 1).

## Discussion

XP is a rare genodermatosis due to a defect of DNA repair resulting in hypersensitivity to sun and ultraviolet rays. Patients with XP present skin changes especially in photo-exposed areas and have an increased risk of cancer. Cutaneous and ocular lesions are the most common clinical symptoms which can be associated to neurological abnormalities.

This autosomal recessive disorder is genetically heterogeneous and due to eight complementation groups with defective NER and a variant group (XP-V) with normal NER. To date, nine genes have been associated with XP: *XPA*, *ERCC3* (*XPB*), *XPC*, *ERCC2* (*XPD*), *DDB2* (*XPE*), *ERCC4* (*XPF*), *ERCC5* (*XPG*), *ERCC1*, and *POLH* (*XP-V*). The diagnosis of XP is based on clinical features. Molecular genetic testing is possible by looking for mutation at one of the causal genes, but this can be achieved only after characterizing the right gene to study by the determination of the complementation group or linkage study in familial cases.

Soufir *et al.* reported the genetic analysis of 66 unrelated Maghrebian families with XP (Algeria, Morocco, and Tunisia) [5]. In this cohort, *XPC* is the major disease-causing gene concerning XP in North Africa with the recurrent mutation c.1643_1644delTG (p.Val548AlafsX25) which is responsible for 74% of all XP [5].

These data allow the molecular diagnosis of Moroccan patients with XP by searching the founder mutation. While sequencing the ninth exon, an insertion of 4-base pairs (4-bp; CATG) between positions 1644_1645 in the complementary DNA (cDNA) was incidentally discovered. This frameshift mutation inducing a stop codon after 25 amino acids is located in the interaction region with RAD23B, which is highly conserved among species according to Mutation Taster Server. Therefore, this variant is very likely the causal mutation of XP in our patient.

**Fig. 2:**
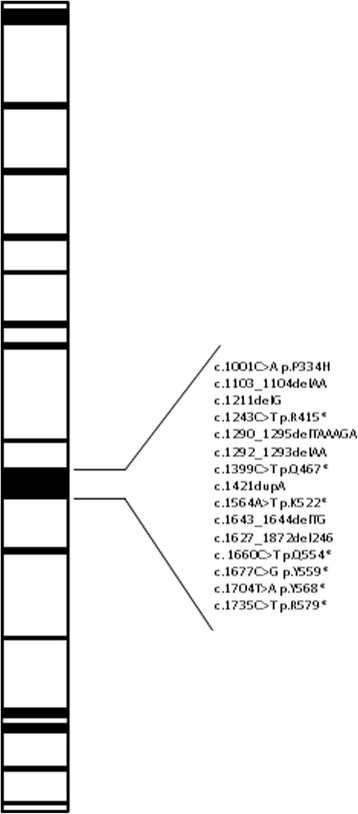
Recorded mutations in exon 9 in te *XPC* gene

About 60 inactivating *XPC* mutations have been reported in patients with XP complementation group C. Most of them are frameshift (deletion and insertion) or nonsense (substitution), leading to a truncated XPC protein. A few splicing and missense mutations are also encountered. According to the Human Gene Mutation Database (HGMD), 25% of all *XPC* gene mutations are located in the ninth exon (Fig. 2). To the best of our knowledge, we report on the result of the first insertion type mutation described within exon 9 whose protein sequence corresponds to the domain of interaction with the RAD23B involved in the NER pathway. These data suggest that sequencing exon 9 could be a first line step in a XP molecular diagnosis strategy in addition to specific founder mutations depending on populations. However, there is no obvious correlation between genotype and phenotype or survival found in XP.

Several studies of population-specific founder mutations have been published. Beside the North African mutation identified in 87% of patients with XP complementation group C [5], other founder mutations were also identified in the Japanese population, one accounting for 90% of alleles in the *XPA* gene [2] and four others representing 87% of XP variant type in Japan [7]. Our patient is a Southern Morocco native and all the reported cases of patients in the previous studies were from North Morocco. Therefore, further patients from South Morocco need to be studied to determine the carrier rate and assess the possibility of a second Moroccan founder mutation.

## Conclusions

Molecular analysis by sequencing the ninth exon of *XPC* gene enables the majority of causal mutations of the disease to be identified, since it comprises the North African founder mutation and almost 25% of the other mutations. This laboratory diagnosis strategy enables the provision of genetic counseling and eventually the prenatal diagnosis of the majority of patients with XP in Morocco, and it enables us to identify a novel mutation whose recurrence would be interesting to study.
